# Residential proximity to agricultural fields, urinary glyphosate levels and breast cancer risk: a case-control study in Argentina

**DOI:** 10.3389/ftox.2025.1579952

**Published:** 2025-05-21

**Authors:** Florencia Doná, Virginia Lorenz, Georgina Stegmayer, Tamara Ricardo, Stefanía D’Iorio, Fernando Ponzo, María Rosa Repetti, Luisina Delma Demonte, María Mercedes Milesi, Jorgelina Varayoud

**Affiliations:** ^1^ Instituto de Salud y Ambiente del Litoral (ISAL), Facultad de Bioquímica y Ciencias Biológicas, Universidad Nacional del Litoral (UNL) - Consejo Nacional de Investigaciones Científicas y Técnicas (CONICET), Santa Fe, Argentina; ^2^ Cátedra de Fisiología Humana, Facultad de Bioquímica y Ciencias Biológicas, Universidad Nacional del Litoral, Santa Fe, Argentina; ^3^ Instituto de Investigaciones en Señales, Sistemas e Inteligencia Computacional, sinc(i), Facultad de Ingeniería y Ciencias Hídricas, UNL, CONICET, Santa Fe, Argentina; ^4^ Departamento de Investigación Epidemiológica, Instituto Nacional de Epidemiología “Dr. Juan H. Jara”, ANLIS Malbrán, Mar del Plata, Argentina; ^5^ Departamento de Matemática, Facultad de Bioquímica y Ciencias Biológicas, UNL, Santa Fe, Argentina; ^6^ Servicio de Ginecología, Hospital José María Cullen, Santa Fe, Argentina; ^7^ Programa de Investigación y Análisis de Residuos y Contaminantes Químicos, Facultad de Ingeniería Química, UNL, Santa Fe, Argentina

**Keywords:** herbicide exposure, urine, breast cancer, case-control study, risk factors

## Abstract

Despite accumulated evidence indicating glyphosate herbicide (GLY) presents endocrine disrupting properties, there are still discrepancies. Moreover, few epidemiological studies have focused on hormone-related pathologies. This work aimed to investigate the associations between urinary GLY levels and breast cancer (BC) in women from a region of intense agricultural activity in Argentina, exploring residential proximity to agricultural fields as a potential risk factor for BC. This was a case-control study that involved 90 women from different populations in the Province of Santa Fe, Argentina. Demographic data, lifestyle factors, and residential history were obtained through a questionnaire, while medical outcomes and reproductive history were acquired from medical records. Spot urine samples were collected and the concentrations of GLY and its primary metabolite, aminomethylphosphonic acid (AMPA) were quantified by ultra-high-performance liquid chromatography–mass spectrometry. Odds ratios were estimated to assess the strength of the association between the case/control type and each predictor. GLY concentrations were above the limit of detection (LOD) in 86.1% of samples, with a range of 0.37–10.07 µg GLY/g creatinine. AMPA was not detected in any of the samples analyzed. Although urinary GLY concentrations showed no differences between the case and control groups, women residing near agricultural fields showed an increased risk of BC (OR: 7.38, 95% CI: 2.74–21.90). These original findings show the ubiquitous presence of GLY in adult women from Argentina. Interestingly, women living near agricultural fields have a higher risk of BC, suggesting that exposure not only to GLY but also to agrochemicals in general, could predispose to the development of BC in Argentina. While this study provides valuable insights, further and broader assessments of BC distribution in relation to agrochemical exposure acroos different regions of Argentina are needed.

## 1 Introduction

In 2022, 2.3 million women worldwide were diagnosed with Breast Cancer (BC), resulting in 670,000 deaths (World Health Organization, 2024). In Argentina, BC is the leading cause of death in women, with 6,100 deaths annually and an estimated 22,000 new cases per year, representing 32.1% of the total cancer incidence (Programa Nacional de Control de Cáncer de Mama (PNCM, 2024). The development of BC is associated with several risk factors, both modifiable and non-modifiable. Among the modifiable risk factors are environmental factors, including chemical agents such as pesticides (Łukasiewicz et al., 2021).

N-(phosphonomethyl)glycine, also known as Glyphosate (GLY), is the active ingredient of commercial formulations named as glyphosate-based herbicides (GBHs). GLY herbicide is a systemic, broad-spectrum and post-emergent agrochemical that is used worldwide for weed control in agriculture, urban and gardening (Acquavella et al., 2004; Andreotti et al., 2018). GLY and its primary metabolite, aminomethylphosphonic acid (AMPA), have been detected in a variety of sources, including water (Aparicio et al., 2013; Bonansea et al., 2017; Mac Loughlin et al., 2017; Singh et al., 2024), soil (Primost et al., 2017; Ayoola et al., 2023), dust resulting from field erosion (Mendez et al., 2017), and food for both human and animal consumption (Rodrigues and De Souza, 2018; Vicini et al., 2021).

Biomonitoring studies have reported the presence of GLY and/or AMPA in urine samples from individuals residing in rural and/or urban areas (Connolly et al., 2022; Chang et al., 2024; Ben Khadda et al., 2025) and, even more worrying, from pregnant and lactating women (Camiccia et al., 2022; Lesseur et al., 2022; Ashley-Martin et al., 2023) and children (Grau et al., 2022; Berni et al., 2023). In addition, GLY concentrations were determined in maternal and umbilical cord serum (Kongtip et al., 2017) and breast milk (Camiccia et al., 2022; Galli et al., 2024). Specifically, in a country with high pesticide use like Argentina, the available data on human exposure to GLY herbicide are limited. To date, concentrations of GLY were documentated only in two regions of Argentina, in the urine of a small rural population from the Province of Chaco (Bressán et al., 2021) and in the urine, plasma, and saliva of an occupationally and non-occupationally exposed male population from the Province of Córdoba (Filippi et al., 2024).

The use of GLY in Argentina began to increase significantly in the mid-1990s, following the introduction of genetically modified GLY resistant soybeans (Benbrook, 2016). This agricultural transition resulted in a substantial increase in GLY application, thereby transforming Argentina into one of the leading consumers of GLY worldwide. Regulatory measures have historically been minimal (Blois and Rendón, 2023); however, growing public concern over health and environmental risks has prompted local-level restrictions and increasing calls for national regulatory reforms, although comprehensive bans or national regulations remain absent (Schmidt et al., 2022).

The utilization of GLY has been extensively implemented throughout the South American continent. In Brazil, a study reported significant contamination of drinking water with GLY and AMPA, particularly in agricultural regions like Paraná, where 100% of the municipalities analyzed exceeded the maximum limits for GLY-AMPA, correlating with increased cancer risks, including BC (Panis et al., 2022). Furthermore, research conducted in Southeast Brazil has revealed the presence of GLY concentrations in drinking water sources reaching up to 8.70 µg/L, thereby exceeding the established safety limits set by national and international regulatory bodies (Lima et al., 2022). In Colombia, in areas dedicated to agricultural activities, the presence of GLY was detected in different water sources near the crops with concentrations ranging from 2.01 to 2.77 µg/L (Alza-Camacho et al., 2016). A recent multicompartmental monitoring study in Uruguay revealed the presence of GLY and other pesticides in the surface water, sediments, and biota of Laguna del Cisne, a subtropical lake utilized as a drinking water source (Rodríguez-Bolaña et al., 2023). The present findings underscore the pervasive environmental occurrence of GLY and underscore the necessity for enhanced surveillance, thereby situating the Argentine context within a more expansive South American framework of pesticide exposure.

Association between GLY exposure and different types of cancer has been researched. Epidemiological studies conducted in Sweden, Canada, and the United States have determined a positive correlation between GLY exposure and the development of non-Hodgkin’s lymphoma (NHL) (Weisenburger, 2021) and multiple myeloma (De Roos et al., 2005). More recently, some authors in a large pooled study did not detect a relationship between GLY (active ingredient) and all types of NHL or multiple myeloma separately, but did find an association with follicular lymphoma, a subtype of NHL (De Roos et al., 2022). Indeed, the carcinogenic potential of GLY has been extensive reviewed and debated by several authoritative and regulatory bodies. The International Agency for Research on Cancer (IARC) classified GLY as ‘probably carcinogenic to humans’ (Group 2A) (EFSA, 2015) based on a specific association with non-Hodgkin’s lymphoma. In contrast to the IARC assessment, the European Food Safety Authority (EFSA) and the Environmental Protection Agency (EPA) concluded shortly after that there was insufficient scientific evidence to consider GLY as possibly carcinogen to humans (Portier et al., 2016; Benbrook, 2019). Despite all this controversy, European Union decided to extend the use of GLY until 2033 (Carrasco Cabrera et al., 2024).

While the debate on its carcinogenic potential continues, increasing evidence indicates GLY as an endocrine disruptor, a chemical with the ability to interfere with hormonal signaling pathways (Muñoz et al., 2021). For instance, GLY has been shown to modulate the activity of sex hormone receptors, particularly the estrogen receptor alpha, by enhancing its transcriptional activation in BC cell lines (Muñoz et al., 2021). Endocrine disruptors, such as pesticides, are of significant concern in BC due to their potential influence on the development and progression of the disease (Wan et al., 2022). In relation to that, a study by Franke et al. (2021) detected higher levels of AMPA in urine samples of women diagnosed with BC compared to controls, suggesting that AMPA exposure may be associated with an increased risk of BC. However, the association between cancer development and GLY exposure in epidemiological studies remains inconclusive. Therefore, further investigations in populations with high GLY exposure, such as those in Argentina, are of great importance.

In the present study, we conducted a case-control study to investigate the associations between urinary GLY levels and BC in women from a region of intense agricultural activity in Argentina, and also exploring residential proximity to agricultural fields as a potential risk factor for BC.

## 2 Methods

### 2.1 Population and data collection

This is a case-control study conducted from January to December 2021. The research protocol was approved by The Research Safety and Ethics Advisory Committee (Code CE2018-50) of the Facultad de Bioquímica y Ciencias Biológicas (Universidad Nacional del Litoral, Santa Fe, Argentina). In the current study, 90 women who attended the gynecology service of José María Cullen Provincial Hospital in Santa Fe were recruited to participate in the study. The study included participants aged 18–60 years old who had lived in various regions of the province of Santa Fe for at least 5 years prior to the interview.

The participants were either diagnosed with BC (cases, n = 30) or were cancer-free patients (controls, n = 60). Prior to their participation, the women were provided with comprehensive written information and were asked to provide their signed informed consent. The data of the participants were obtained from their medical records and survey responses. Since not all women completed the questionnaire, sample size for some variables was less than 30 in the case group and less than 60 in the control group. The questionnaire included items pertaining to demographic data, health information, lifestyle factors, reproductive history, family history of BC, and occupational risk factors. The participants’ addresses were obtained from medical records and georeferenced using Google Maps (Online Geocoder). The resulting coordinates were then classified based on the proximity to agricultural fields (as residential proximity). Addresses located within 4,000 m were considered ‘near’, while those located beyond 4,000 m were considered ‘far’ (Thompson et al., 2022). Out of 90 women surveyed, urine samples were collected from 70 patients (16 BC cases and 54 controls). For the remaining 20 patients, the conditions for urine sampling could not be met since women were menstruating or lacked the minimum retention time. The samples were transported to the Instituto de Salud y Ambiente del Litoral (UNL-CONICET) for processing on the day of collection. All samples were de-identified, stored at −80°C, and then shipped to the laboratory of the Programa de Investigación y Análisis de Residuos y Contaminantes Químicos (PRINARC) for analysis of GLY and AMPA concentrations.

### 2.2 Creatinine analysis

The urine samples were analyzed for creatinine by a colorimetric method using the Wiener Lab Creatinine Kit (code 1260001, Rosario, Argentina). The reaction of creatinine with alkaline picrate in a buffered medium results in the formation of a chromogen, which is then measured at 510 nm. The purpose of this analysis is to ascertain the validity of the samples and to normalize for urine concentration. According to the World Health Organization (World Health Organization, 1996), samples with creatinine levels below 30 mg/dL or above 300 mg/dL should be discarded. Samples with very low creatinine concentrations may interfere with the detection of low-level toxicants, while those with very high creatinine concentrations may indicate dehydration, which may affect the renal secretion, excretion, and/or reabsorption of target chemicals (Barr et al., 2005).

### 2.3 Urinary GLY and AMPA analyses

#### 2.3.1 Standards and reagents

Crystalline standards of GLY (97%), AMPA (98%), GLY-9-fluorenylmethylchloroformate (GLY-FMOC) (92%) and AMPA-FMOC (98%) were from Dr. Ehrenstorfer (Augsburg, Germany), and the GLY Isotope-Labeled Internal Standard 1,2-^13^C_2_^15^N (98%) was obtained from Toronto Research Chemicals (Toronto, Canada). Solutions of the derivatizing reagent FMOC-Cl (Sigma, St. Louis, MO, United States) and sodium tetraborate buffer (Anedra, San Fernando, Buenos Aires, Argentina) were separately prepared by dissolving the reagents in acetonitrile and water, respectively. Ultra-high performance liquid chromatography (UHPLC)-grade acetonitrile and methanol (OptimaTM, Fisher Scientific, NJ, United States) and deionized water produced with a Milli-Q System (Millipore, Bedford, MA, United States) were utilized for mobile phase preparation. Additionally, 5 mM ammonium acetate (Anedra, San Fernando, Buenos Aires, Argentina) was employed as modifier to promote ionization. Pesticide grade dichloromethane (Sintorgan, Buenos Aires, Argentina) was used for clean-up purposes.

#### 2.3.2 Analytical method

Human urine samples were analysed for the presence of GLY and AMPA. Sample preparation was adapted from Bernal et al. (2010), with derivatisation based on the method outlined by Demonte et al. (2018), as described below. In an eppendorf tube, 500 μL of human urine was spiked with 20 μL of GLY 1,2- ^13^C_2_^15^N, followed by the addition of 250 μL of acetonitrile. The mixture was vortexed for 1 min, subjected to 10 min of ultrasound treatment, and centrifuged at 15,000 rpm for 15 min at room temperature to precipitate proteins. Subsequently, 500 μL of the supernatant was transferred to another eppendorf tube, and the protein precipitation step was repeated once more. The supernatant (500 μL) underwent derivatisation by adding 84 μL of borate buffer (40 mM, pH 9) and 84 μL of FMOC-Cl. The concentration of FMOC-Cl was adjusted based on the creatinine content of the original sample. The reaction was allowed to proceed for 2 h at room temperature. Following derivatisation, the extracts were cleaned by liquid-liquid partition with 500 μL of dichloromethane. Finally, a fraction of the aqueous phase was injected into the UHPLC-MS/MS system.

Quality assurance and quality control (QAQC) procedures were implemented as follows. Due the unavailability of blank samples, samples tested without GLY were used as blank samples and each urine sample was spiked with internal standard. Duplicates of each sample were spiked with GLY-FMOC and AMPA-FMOC standard solution to achieve three different concentration levels: one-third of the samples were spiked at 0.50 μg/L, one-third at 1.00 μg/L, and the remaining at 5.00 μg/L.

Percentage recoveries ranged from 62% to 126%, with a relative standard deviation (RSD) lower than 25% for all samples and both compounds. The limit of detection (LOD) and the limit of quantitation (LOQ) were determinate using S/N ratios of 3 and 10, respectively, from 1.00 μg/L spiked samples chromatogramas. LOD were 0.10 μg/L for GLY and AMPA. The LOQs (0.50 μg/L for GLY and AMPA) were experimentally verified by analyzing spiked samples at LOQ level, with recoveries and RSD within acceptable ranges.

To assess linearity, isotopically labelled GLY and AMPA standard working solution was added to urine samples with varying creatinine contents: 31.30 mg/dL (low level), 153.05 mg/dL (medium level), and 300.00 mg/dL (high level). Since AMPA was not detected in samples, the matrix effect was assessed solely by analyzing six standard solutions of GLY 1,2-^13^C_2_^15^N in triplicate within the range of 0.10–25.00 μg/L (0.10, 0.50, 1.00, 5.00, 10.00, 25.00 μg/L). As it was demonstrated that the matrix effect depends on the level of creatinine in the sample, the concentration of GLY was calculated with the internal standard added to each sample.

#### 2.3.3 Chromatographic system and operating conditions

Liquid chromatography with tandem mass spectrometry (LC-MS/MS) analyses were performed using an Acquity UPLC^TM^ liquid chromatograph (Waters, Milford, MA, United States) coupled to a triple quadrupole mass spectrometer equipped with an electrospray ionization source able to operate in positive and negative-ion mode (TQD, Waters Micromass, United Kingdom).

Chromatographic separation was evaluated on an X-Select CSH-C18 column (3.50 μm particle size, 100.00 × 4.60 mm i.d) at 40°C. Aliquots of 10 μL of standard and/or sample extracts were introduced by means of an auto-sampler (Waters, Milford, MA, United States).

The mobile phase consisted of water and acetonitrile (98:2) + 5 mM NH4Ac (solvent A) and acetonitrile (solvent B). Chromatographic and mass spectrometry data were handled using MassLynx software v 4.1 (Waters, Manchester, United Kingdom).

All these procedures were performed in the PRINARC at the Facultad de Ingeniería Química, Universidad Nacional del Litoral, Santa Fe, Argentina.

### 2.4 Data analysis

#### 2.4.1 Statistical analysis

Statistical analysis was performed using R software (version 4.2.0). Urinary herbicide concentrations were reported as µg/g creatinine (creatinine-adjusted concentrations). A descriptive analysis of the surveyed variables was performed showing medians and interquartile ranges (IQR) for continuous variables, or frequencies and percentages for categorical variables, as appropriate. The continuous variables included age, weight, height, and body mass index (BMI) calculated from the latter two. Categorical variables were grouped into different dimensions: pesticide exposure (e.g., living near agricultural fields, childhood in rural areas, rural work, use of agrochemicals, family members working with pesticides, pesticide application at home), lifestyle factors (e.g., smoking, alcohol consumption, physical activity, consumption of vegetables, fruits, dairy products, processed meats, red meat, white meat, drinking water source), and socioeconomic status (e.g., educational level, employment status).

Due to the non-normal distribution of the data, non-parametric bivariate tests were used. Each BC patient was matched with two controls based on age, residence proximity classified based on the proximity to agricultural fields and sample provision. Associations between BC and each covariate were initially assessed using the Mann-Whitney U test or Kruskal-Wallis test for continuous variables, and Pearson’s χ2 or Fisher’s exact tests for categorical variables, to identify possible candidate variables for multivariate logistic regression. Variables with a p-value < 0.10 were included in the multivariable logistic regression model. In this model, the response variable was of the case/control type, whereby cases were assigned the value of ‘1’ and controls the value of ‘0’. Odds ratios were estimated with a 95% confidence interval (CI) to evaluate the strength of the association between the response variable and each predictor.

#### 2.4.2 Supervised machine learning

Supervised learning is a machine learning (ML) paradigm in which the data set comprises labeled examples. Each data point contains features and an associated output label. The goal of supervised learning algorithms is to learn a function that maps feature numerical vectors (inputs) to labels (desired outputs) based on example input-output pairs (Russell and Norvig, 2016) also known as training examples (Bishop and Nasrabadi, 2006). A supervised learning algorithm analyzes the training data and produces an inferred function that can be used to map new testing examples, generalizing from the training data to unseen situations.

In this study, several ML classifiers were trained, including Multilayer Perceptron (MLP), Random Forest (RF), Gradient Boosting (Gboost), Bagging and K nearest neighbors (KNN) (Haykin, 1999; James et al., 2013). We have also included a logistic regression linear model as a benchmark for comparison with the machine learning models. The optimal model was selected using random cross-validation, with 1-fold completely random partition having 80% of the complete dataset allocated for training and 20% of the full dataset for testing and performance evaluation. For the MLP model, hyperparameters grid-search was performed with a small subset of the training data. The MLP architecture is hidden_layer_size = 100, activation function = reLU, automatic batch_size, learning_rate = 0.001, and maximum iteration number = 1,000. The optimizer employed was Adam. The other models were used with default parameters: RF, GBoost and Bagging with 100 estimators, KNN with k = 5.

The prediction quality of each model was evaluated using the F1 classical classification measure:

$$F_{1}=2\frac{s^{+}p}{s^{+}+p},s^{+}\frac{TP}{TP+FN},p\frac{TP}{TP+FP}$$

where s+ (recall) measures how good is a classification method at recognizing (and not missing) the true positives; the precision p measures the relation between true positives and false positives; F1, a harmonic score between precision and recall, is used to compare prediction methods. TP, FP and FN represent the number of true positive, false positive and false negative classifications, respectively.

Model explainability, named Explainable Artificial Intelligence (XAI) is crucial in any ML pipeline. In this work, XAI with SHAP (Shapley Additive Explanations) was employed to achieve model explainability. This work used the SHAP method (Lundberg and Lee, 2017), which is based on the game theoretically optimal Shapley values (Shapley, 1953), to explain individual predictions by computing the contribution of each feature to the prediction.

The SHAP explanation method utilizes coalitional game theory to compute Shapley values. In this method, the feature values of a data instance act as players in a coalition. Shapley values explain how to distribute the prediction playout fairly among the features. In the case of tabular data, a player can be an individual feature value. SHAP specifies the explanation as:

$$g\left(x^{\prime }\right)=\emptyset_{0}+\sum_{j=1}^{M}\emptyset_{j}$$

where *g* represents the explanation model, *x’* represents an instance o data point, *M* represents the number of features and *ϕj∈R* represents the feature attribution for a feature *j*, also known as the values. The Shapley value for feature j indicates the value contributed by the j-th feature to the prediction output of this particular instance compared to the average prediction for the dataset.

In this study, we used the SHAP Summary Plot to show the importance of each feature for the RF model trained to predict cancer. In this plot, the effect of a feature on the classes is stacked to create the feature importance plot. The summary plot for multiclass classification can show what the model learns from the features. Each point on the summary plot represents a Shapley value for a feature and an instance. The y-axis position corresponds to the feature, and the x-axis position is determined by the Shapley value. The color represents the value of the feature from lowest (red) to highest (green) value. The features are ordered according to their importance. Additionaly, we obtained a detailed SHAP Summary plot for each class.

## 3 Results

### 3.1 Population characteristics

**FIGURE 1:**
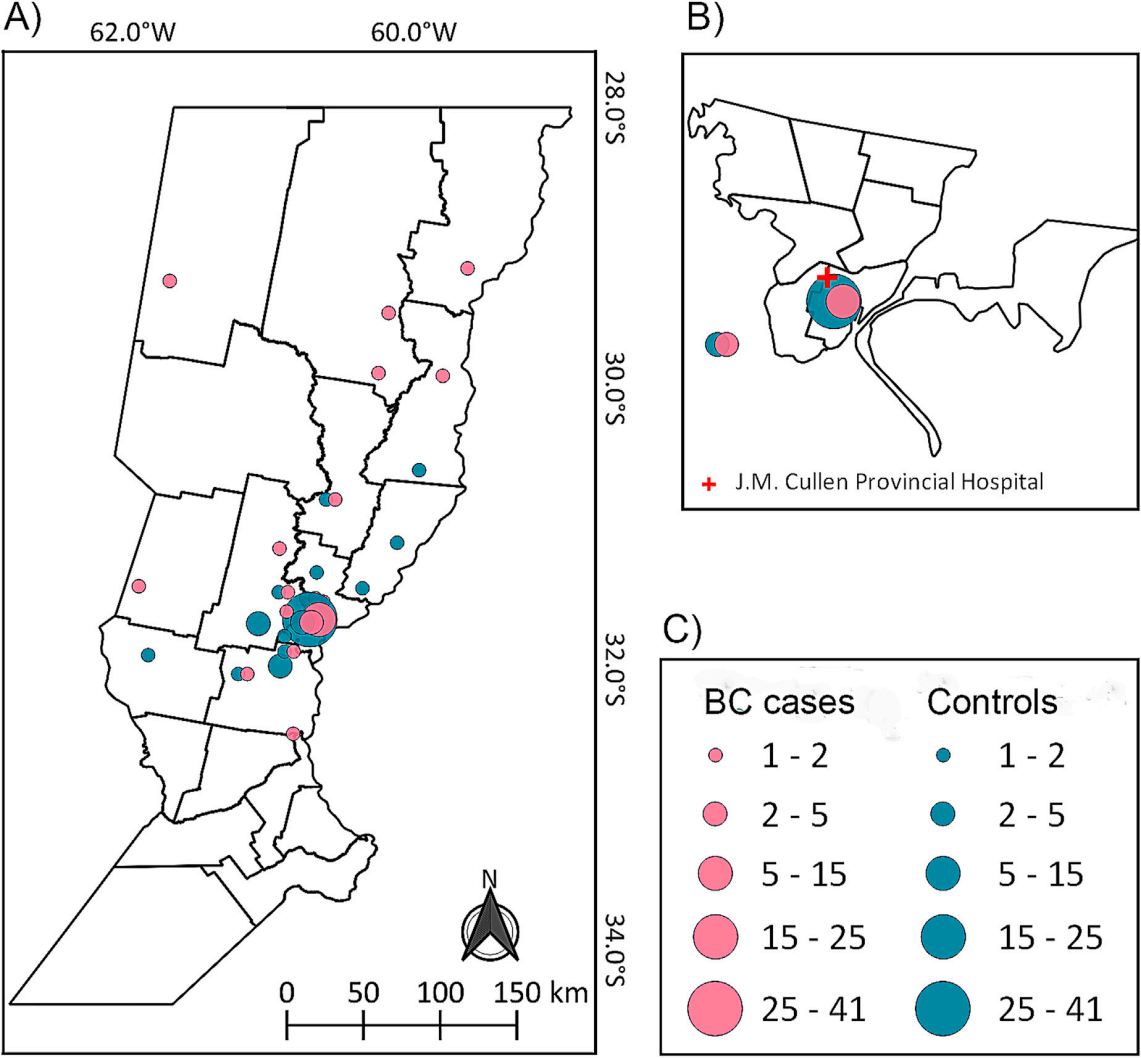
**(A)** Map of the province of Santa Fe showing the geographic locations of the women’s residences. Color represents both BC cases (pink) and the control group (blue). **(B)** The expanded area of the district of Santa Fe city. José María Cullen Provincial Hospital, where the patients attended for medical attention, is indicated with a red cross. **(C)** Plot sizes are proportional to the number of participants. The map figure was created using QGIS software version 3.22.15 (QGIS Development Teaikm). Vector layers were provided by the Instituto Geográfico Nacional (IGN) and the Gobierno de la Provincia de Santa Fe.

The questionnaire was completed by 30 BC cases and 60 controls. Figure 1 indicates the geographic locations of the women’s residences. General characteristics of case and control groups are shown in Table 1 and their dietary habits are displayed in Supplementary Table S1 (Supplementary Material). The control group had a median age of 42 years old (interquartile range (IQR): 36–50 years), while the BC group had a median age of 48 years old (IQR: 41–54 years). Body mass index (BMI) was not significantly different between the groups, with a median of 28.6 (IQR: 23.8–33.5) kg/m^2^ for controls and a median of 26.7 (IQR: 24.3–30.3) kg/m^2^ for cases (p = 0.409). Educational level was not significantly different between BC cases and controls (p = 0.467), with only 37.9% of BC cases and 40.7% of controls having completed secondary school. Menopausal status was significantly different between BC cases and controls (p = 0.028). The majority of participants, both cases (56.7%) and controls (61.7%) reported never having had a mammogram. Also, no significant differences were detected between the cases and controls with regard to the age at menarche, family history of BC, oral contraceptive use, hormone replacement therapy, breastfeeding, physical activity, smoking, alcohol consumption, and meat and vegetable intake. Table 2 shows the data on the participants’ exposure to pesticides. In this study, 66.7% of the BC cases and 23.3% of controls reported residing near an agricultural fiel (defined as residential proximity) (p < 0.001).

**TABLE 1:** Characteristics of case and control women living in the city of Santa Fe and surroundings.

Variables + numeric encoding	Cases N (%) or median [IQR]	Controls N (%) or median [IQR]	P-value
Age (years)	48 [41.20–53.80]	42 [36.00–50.00]	0.086
BMI (kg/m^2^)			0.247
Normal weight (18.50–24.90) (0)	9 (30.0%)	20 (33.3%)	
Overweight (25.00–29.90) (1)	13 (43.3%)	16 (26.7%)	
Obesity (30.00 or higher) (2)	8 (26.7%)	24 (40.0%)	
Educational level			0.467
Incomplete primary education (0)	2 (6.9%)	1 (1.7%)	
Complete primary education (1)	16 (55.2%)	34 (57.6%)	
Complete secondary education (2)	11 (37.9%)	24 (40.7%)	
Working status			0.248
No (0)	16 (59.3%)	25 (43.1%)	
Yes (1)	11 (40.7%)	33 (56.9%)	
Age at menarche (years)			0.355
<12 (0)	17 (56.7%)	29 (48.3%)	
12–14 (1)	8 (26.7%)	25 (41.7%)	
>14 (2)	5 (16.6%)	6 (10.0%)	
Age at first live birth			0.368
<30 years	23 (76.7%)	52 (86.7%)	
≥30 years	7 (23.3%)	8 (13.3%)	
Number of children			0.747
0	2 (6.7%)	4 (6.7%)	
1	4 (13.3%)	9 (15.0%)	
2–3	16 (53.3%)	25 (41.7%)	
4	8 (26.7%)	22 (36.6%)	
Breastfeeding			1.000
No (0)	1 (3.6%)	2 (3.6%)	
Yes (1)	27 (96.4%)	53 (96.4%)	
Breastfeeding duration (months)	21.00 [11.50–24.00]	24.00 [12.00–24.00]	0.441
Oral contraceptive use			0.697
No (0)	12 (40.0%)	20 (33.3%)	
Yes (1)	18 (60.0%)	40 (66.7%)	
Menopausal status			0.028
Premenopausal (0)	16 (53.3%)	47 (78.3%)	
Postmenopausal (1)	14 (46.7%)	13 (21.7%)	
Hormone replacement therapy			1.000
No (0)	29 (96.7%)	57 (95.0%)	
Yes (1)	1 (3.3%)	3 (5.0%)	
Mammography screening			0.819
No (0)	17 (56.7%)	37 (61.7%)	
Yes (1)	13 (43.3%)	23 (38.3%)	
Family history of BC			0.216
No (0)	27 (90.0%)	46 (76.7%)	
Yes (1)	3 (10.0%)	14 (23.3%)	
Tobacco consumption			0.936
No (0)	20 (66.7%)	42 (70.0%)	
Yes (1)	10 (33.3%)	18 (30.0%)	
Alcohol consumption			0.800
No (0)	23 (76.7%)	43 (71.7%)	
Yes (1)	7 (23.3%)	17 (28.3%)	
Drinking water source			0.083
Potable tap/mineral water (0)	12 (75.0%)	39 (92.9%)	
Non-potable well water (1)	4 (25.0%)	3 (7.1%)	
Physical activity			0.341
<2 h per week (0)	9 (56.2%)	16 (38.1%)	
≥2 h per week (1)	7 (43.8%)	26 (61.9%)	
Vitamins			0.672
No (0)	13 (81.2%)	37 (88.1%)	
Yes (1)	3 (18.8%)	5 (11.9%)	

**TABLE 2:** Data on pesticide exposure of case and control women in the city of Santa Fe and surroundings.

Variables + numeric encoding	Cases N (%) or median [IQR] (ND: Not detected)	Controls N (%) or median [IQR] (ND: Not detected)	P-value
Urinary levels of GLY (µg GLY/g creatinine)	0.30 [0.25–0.36]	0.33 [0.17–0.86]	0.395
Urinary levels of AMPA	ND	ND	
Residence near agricultural fields			<0.001
No (0)	10 (33.3%)	46 (76.7%)	
Yes (1)	20 (66.7%)	14 (23.3%)	
Rural labor tasks			0.097
No (0)	11 (68.8%)	38 (90.5%)	
Yes (1)	5 (31.2%)	4 (9.5%)	
Use of agrochemicals			0.696
No (0)	13 (81.2%)	36 (85.7%)	
Yes (1)	3 (18.8%)	6 (14.3%)	
Application of pesticides at home			0.720
No (0)	12 (75.0%)	34 (81.0%)	
Yes (1)	4 (25.0%)	8 (19.0%)	
Family members working with pesticides			0.030
No (0) Yes (1)	11 (68.8%) 5 (31.2%)	39 (92.9%) 3 (7.1%)	

Urinary creatinine analysis showed 65 out of 70 samples were valid (14 BC cases and 51 controls) with creatinine values ranging 30–300 mg/dL. GLY was detected in 86.1% of the 65 urine samples tested. Concentrations ranged 0.37–10.07 µg GLY/g creatinine and no significant differences were observed between the BC cases and controls. AMPA was not detected in the samples tested.

The results of the multivariate logistic regression analysis showed a significant increase in the risk of BC for women residing near agricultural fields (OR: 7.38, 95%CI: 2.74-21.90) and for postmenopausal status (OR: 3.80, 95% CI: 1.32-11.70). None of the associations found were related to urinary GLY levels.

### 3.2 ML analysis

The dataset comprises 90 women, with 30 cases of BC and 60 controls. The target class, ‘Group’, indicates whether the participant has been diagnosed with BC (case group) or not (control group). Table 1 presents the description and details of each dataset feature used in this study. Binary variables (the ‘Yes/No’ type) were assigned values of 0 (No) and 1 (Yes). Additionally, variables with more than two levels were coded incrementally to indicate their respective levels.

In the cross-validation test, each model achieved the following F1 scores at the 20% data test partition: logistic regression 66.7%, MLP 61.1%, RF 77.8%, GBoost 72.2%, Bagging 61.1% and KNN 61.1%. Therefore, RF was selected as the best model for cancer prediction to perform the final training with the complete dataset.

**FIGURE 2:**
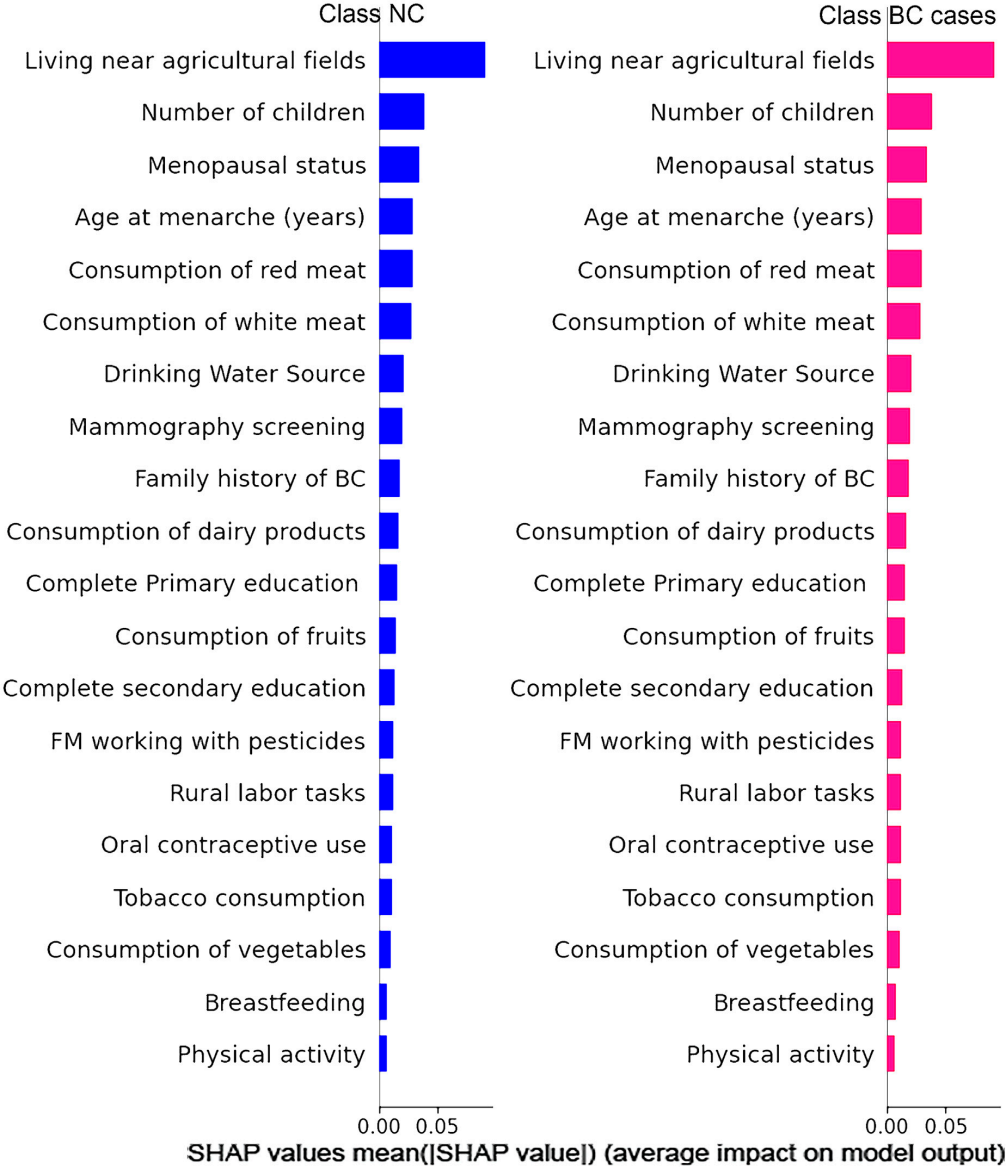
The SHAP summary bar graph shows the ranking of variable importance based on the mean absolute value (|SHAP value|) for both controls (Class NC) (blue) and BC cases (Class BC cases) (pink). FM: Family members.

**FIGURE 3:**
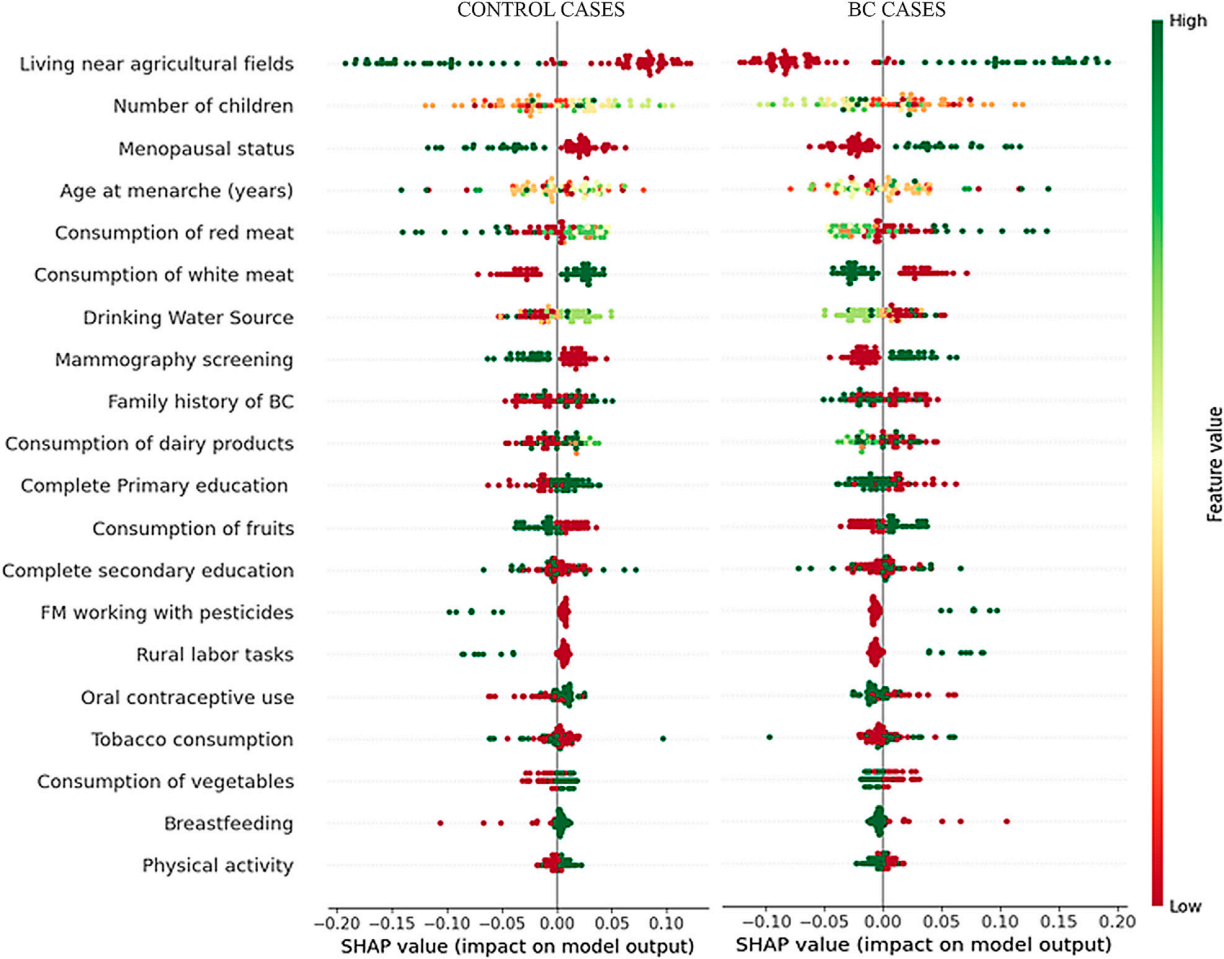
The SHAP summary plot for each class: control cases (left) and BC cases (right) in detail. The y-axis position corresponds to the feature, and the x-axis position is determined by the Shapley value. The color indicates the value of the feature, ranging from lowest (red) to highest (green) value. SHAP values above 0 indicate a positive association with the outcome (for example, regarding BC cases, high values of the feature “living near agricultural fields” have a positive impact on the output of the model for this class). SHAP values below 0 indicate a negative association with the outcome (for example, regarding BC cases, low values of the feature “living near agricultural fields” have a negative impact on the output of the model for this class).

After the cross-validation that was performed in order to select the best classifer, a RF classifier of 100 trees was trained with the complete dataset (no partitions) to classify instances into two classes: ‘no-cancer’ (Class NC) and ‘BC’ (Class BC cases), resulting in F1 = 68.6% for the complete dataset. The SHAP method was then employed to explain the results of the RF model. The features were ranked based on their importance, as shown in Figure 2. The SHAP bar graph shows the mean absolute SHAP values, which represents the average impact of each feature on the model’s predictions for each class. The most important feature for classification into these classes is living near agricultural fields. Figure 3 show the SHAP plots illustrating the impact of each feature on the model output for each separate class. It was observed that for class BC, the variable of ‘living near agricultural fields’ has a very high impact on the model output.

## 4 Discussion

The present study, similar to others conducted in different countries, demonstrated the ubiquitous presence of urinary GLY in women from Santa Fe, Argentina. The widespread presence of GLY in human samples reflects its pervasive occurrence in the environment, as GLY have been reported in various food matrices (Zoller et al., 2018; Fagan et al., 2020; Cruz and Murray, 2021) and, particularly in Argentina, in different environmental samples (Primost et al., 2017; Lutri et al., 2020; Okada et al., 2020). Moreover, a recent study examining the occurrence of pesticides in 64 small water bodies across regions with intense agricultural activity in Argentina and ten European countries revealed that GLY herbicide exhibited the highest median concentration (Navarro et al., 2024). Even more worrying, Argentina showed the highest overall concentration of pesticides in the water bodies analyzed (Navarro et al., 2024). This evidence suggests a significant burden of GLY exposure in the Argentine population.

In our study, the median GLY concentration, including both healthy subjects and those diagnosed with BC, was 1.23 μg/L, with a maximum of 3.50 µg/L observed in a healthy woman. These urinary GLY levels were higher compared to other studies carried out in Argentina in non-occupationally exposed men (median 0.191 ng/mL) (Filippi et al., 2024) and in a population from a rural village (range < 0,50–3,03 μg/L) with 19.2% of quantifiable samples (Bressán et al., 2021). Moreover, the GLY levels reported in the current work were also higher than those in healthy postmenopausal women in Southern California, United States (median 0.10 μg/L; maximum 3.01 μg/L) (Lucia et al., 2023), healthy lactating women in the United States (mean 0.28 μg/L; range 0.02–1.93 μg/L) (McGuire et al., 2016), and adults in Portugal (median 0.13 μg/L) (Nova et al., 2020). On the other hand, some authors detected similar maximum urinary GLY concentrations of 3.22 μg/L in Danish mothers (Knudsen et al., 2017) and 3.39 μg/L in Swedish young adults (Faniband et al., 2021). To better contextualize GLY and AMPA exposure levels across regions, Table 3 presents a comparative summary of GLY and AMPA levels reported in biological and environmental samples from Argentina, neighboring South American countries, and Europe.

**TABLE 3:** GLY and AMPA concentrations detected in biological and environmental samples from South American and European countries.

Type of sample	Results	Detection methods	Country	Authors	DOI
Human urine (27 female/25 male from Chaco lived in a small rural village)	Only 10 samples (19.2%) showed quantifiable values (median: 0.30 µmol/mol creatinine; range: (0.12–0.91) µmol/mol creatinine)	Liquid chromatography coupled to tandem mass spectrometry (LC-MS/MS)	Argentina	Bressan et al. (2021)	https://doi.org/10.1016/j.jchromb.2021.122616
Human urine (15 subjects occupationally and 20 environmentally exposed to pesticides)	Urine of non-occupationally exposed population: AMPA: median 0.27 ng/mg creatinine, GLY median 0.10 ng/mg creatinine Urine from occupationally exposed population. AMPA: median 0.38 ng/mg creatinine, GLY median 0.08 ng/mg creatinine	Gas chromatography coupled to tandem mass spectrometry (MS/MS)	Argentina	Filippi et al. (2024)	https://doi.org/10.1016/j.envadv.2023.100474
Human urine (90 farmers)	12% of the farmers presented GLY levels	High-performance liquid chromatography (HPLC-FL)	Brazil	de Melo et al. (2020)	https://doi.org/10.31005/iajmh.v3i0.124
Human urine (519 participants lived in agricultural communities)	GLY was detected in 98.3% of participants Geometric mean (95% IC) 0.92(0.83,1.01)	Isotope-dilution mass spectrometry	Ecuador	Chronister et al. (2023)	https://doi.org/10.1289/EHP11383
Human urine (French general population)	GLY quantifable in 99.8% of urine samples with a mean of 1.19 ng/mL+/−0.84 after adjustment to body mass index (BMI)	ELISA	France	Grau et al. (2022)	https://doi.org/10.1007/s11356-021-18110-0
Human urine (non-farm and farm families)	GLY (max) 3.21 µg/L, AMPA (max) 7.24 µg/L	GC–MS/MS	Ireland	Connolly et al. (2022)	https://doi.org/10.3390/tóxicos10110690
Human urine (Young adults (18–19 years old)	The median concentration was below 0.10 μg/L and a maximum concentration being 3.39 μg/L (density adjusted)	LC-MS/MS	Sweden	Faniband et al. (2021)	https://doi.org/10.1016/j.ijheh.2020.113657
Surface waters, raw water and drinking water	Surface waters: GLY: range LOQ (0.25 µg/L) – 0.50 µg/L, AMPA: range LOQ (0.67 µg/L – 0.70 µg/L) In samples of raw water and drinking water the results for GLY and AMPA could not be quantified	Enzyme-Linked Immunosorbent Assay (ELISA)	Uruguay	Frontera et al. (2024)	https://doi.org/10.26461/27.01
Water	GLY was detected in 66% of surface water samples (0.20–167.40 μg/L), in 15.8% of the groundwater samples (1.30–2.00 μg/L) and in the harvested precipitation sample (0.20 μg/L)	UHPLC MS/MS	Argentina	Lutri et al. (2020)	https://doi.org/10.1016/j.scitotenv.2019.134557
Soil	The average concentrations of GLY and AMPA in soil were 2,299 ± 476 mg/kg and 4,204 ± 2,258 mg/kg, respectively	Ultra-performance liquid chromatography with tandem mass spectrometry (UPLC-MS/MS)	Argentina	Primost et al. (2017)	https://doi.org/10.1016/j.envpol.2017.06.006
Rainwater	Maximum detected concentrations were 6.10 μg/L and 5.80 μg/L for GLY and AMPA, respectively	LC–MS/MS	Belgium	Tang et al. (2015)	https://doi.org/10.1016/j.scitotenv.2015.02.040

In our study, the metabolite AMPA was not detected in any of the urine samples analyzed, which differs from other studies that have reported its presence. This discrepancy could be attributed to a number of factors including limited exposure, removal efficiency or limitations in the detection method (Aris and Leblanc, 2011). Our method achieved a LOD of 0.10 µg/L for AMPA, which is comparable to those reported in other biomonitoring study (Faniband et al., 2021). In that study, AMPA was detected in a higher percentage of urine samples than GLY; however, no significant correlation was observed between urinary concentrations of GLY and AMPA. This finding suggests that AMPA levels in urine may not necessarily reflect internal GLY metabolism but could instead be influenced by independent environmental exposure to AMPA. Futhermore, only about 1% of an ingested GLY dose is excreted unchanged in urine, with reported excretion rates for AMPA being even lower. These biological factors may explain the absence of AMPA in urine, particularly when exposure originates primarily from GLY.

Some studies have shown a correlation between pesticide exposure and increased BC risk in vulnerable populations (Franke et al., 2021; De Rezende et al., 2023; Panis et al., 2024). For instance, a case-control study in Paraná, Brazil, a region with extensive pesticide use, examined the impact of pesticide exposure on BC risk in rural women who performed cleaning tasks on pesticide-contaminated equipment and clothing (Panis et al., 2024). The authors found evidence of pesticide exposure, including GLY in urine samples, and revealed that women exposed to pesticides exhibited an elevated risk of BC and lymph node metastasis (Panis et al., 2024). In contrast, in our current study we did not find a direct association between the urinary GLY levels and BC risk. This finding suggests that the relationship between urinary GLY concentration and BC risk may be complex and influenced by additional factors beyond just GLY exposure. Furthermore, taking into account the ubiquitous occurrence of GLY in our healthy and unhealthy population, make it more difficult to establish an association between the levels of this herbicide and the risk of BC and other pathologies.

Importantly, the evaluation of residential proximity revelated that women residing near agricultural fields had increased risk of BC, a finding consistent across both clasiccal statistics and ML approach. This result aligns with previous studies reporting increased BC risk associated with proximity to agricultural areas and surrounding greenness in Spain (O’Callaghan-Gordo et al., 2018). Additionally, a case-control study conducted also in Spain revelead that children residing near agricultural areas had a higher risk of developing various types of cancer (Gómez-Barroso et al., 2016). In France, Carles et al. (2017) found an increased risk of meningioma among adults residing near open field crops. Despite the absence of an association between BC and urinary GLY levels in our study, the positive correlation detected between BC and proximity to cultivated fields suggests that exposure to agrochemicals in general, and not just GLY, may contribute to BC development. While our results are consistent with these previous studies, it is important to note that the existing literature is not yet sufficiently conslusive. This is due, in part, to the heterogeneity in study desings, with some focusing on different cancer types or employing various methods to assess pesticide exposure.

Another interesting finding of this work was the detection of an increased risk of BC in postmenopausal women. This result is consistent with previous data indicating that the majority of BC diagnoses occur in postmenopausal women (Kharb et al., 2020). The postmenopausal period is considered a particularly susceptible phase to the influence of endocrine-disrupting chemicals (EDCs), and growing evidence suggests that EDCs exposure may contribute to BC development, especially during this physiological period (Kass et al., 2020; Gouesse and Plante, 2022).

Notable, GLY has shown estrogenic properties and the ability to induce the proliferation of estrogen-dependent MCF-7 human BC cells (Thongprakaisang et al., 2013; Mesnage and Antoniou, 2017) and ERalpha (ESR1)-positive cholangiocarcinoma cells (Sritana et al., 2018). *In vivo* studies have also demonstrated GLY’s potential to induce hyperplastic changes in the mammary gland of aged female rats (Zanardi et al., 2020). Based on this evidence, GLY herbicide might be a contribuiting factor in the development of BC. However, further epidemiological research is necessary to address this point, with particular attention to menopausal status.

This study provides evidence for the widespread presence of GLY in urine samples from women residing in Santa Fe, Argentina. Despite the absence of a direct association between GLY levels and BC, we detected a significant association between BC risk and living near agricultural fields. Our findings highlight the importance of considering environmental factors, including pesticides, when assessing BC risk. Finally, this work underscores the need to develop strategies to reduce pesticide exposure and protect the health mainly of those populations living near agricultural fields.

### 4.1 Limitations and strengths of the study

Our study has limitations, mainly because of the small number of participants, which requires further research with a larger population to increase the power of the study and to better explore these relationships while accounting for relevant confounding factors such as, age, menopause status and family members working with pesticides. Another limitation is that our case-control ratio does not reflect the actual disease incidence in the general population due to recruitment constraints, which could potentially introduce selection bias in our analyses. The limited sample size prevented us from properly stratifying participants by menopausal status, which would be important in future studies given its established association with BC risk. Additionally, the utilization of a single urine sample may not be sufficient to accurately assess GLY exposure levels, given the short half-life of this compound. Nevertheles, our findings indicate that GLY is present in most of the samples analyzed, suggesting the widespread occurrence in the population. Furthermore, the results provide substancial insights into potential risk factors for BC in our population, particularly related with the place of residence. In addition, menopause may represent a vulnerable physiological period associated with increased BC risk in our population. We consider the data from the present study to be of significant importance for future decision-making in Argentina.

## Data Availability

The original contributions presented in the study are included in the article/Supplementary Material, further inquiries can be directed to the corresponding author.

## Abbreviations

AMPA, aminomethylphosphonic acid; BC, Breast Cancer; BMI, body mass index; CI, confidence interval; EFSA, European Food Safety Authority; EPA, Environmental Protection Agency; GLY, glyphosate; IARC, International Agency for Research on Cancer; IQR, interquartile ranges; ML, machine learning; OR, Odds ratios; UHPLC-MS/MS, Ultra high-performance liquid chromatography–Mass spectrometry.
