# Supplementary material for: Residential proximity to agricultural fields, urinary glyphosate levels and breast cancer risk: a case-control study in Argentina
Source: Front Toxicol. 2025 May 21;7:1579952. doi: 10.3389/ftox.2025.1579952 (PMC12133821; doi:10.3389/ftox.2025.1579952)
Supplement: Supplementary file 1 [file Table1.docx]

**Supplementary Information**

**TABLE S1** Dietary habits of cases and controls women living in the city of Santa Fe and surroundings.

| Variables + numeric encoding | **Cases**  N (%) or median [IQR] | **Controls**  N (%) or median [IQR] | P-value |
| --- | --- | --- | --- |

| **Consumption of vegetables** |  |  | 0.660 |  |
| --- | --- | --- | --- | --- |
| Once a day or less (0) | 1 (6.25%) | 6 (14.3%) |  |  |
| Twice daily or more (1) | 15 (93.8%) | 36 (85.7%) |  |  |
| **Consumption of fruits** |  |  | 0.044 |  |
| Once a day or less (0) | 2 (12.5%) | 19 (45.2%) |  |  |
| Twice daily or more (1) | 14 (87.5%) | 23 (54.8%) |  |  |
| **Consumption of dairy products** |  |  | 0.259 |  |
| Once a day or less (0) | 1 (6.25%) | 10 (23.8%) |  |  |
| Twice daily or more (1) | 15 (93.8%) | 32 (76.2%) |  |  |
| **Consumption of processed meats** |  |  | 0.149 |  |
| No (0) | 1 (6.25%) | 11 (26.2%) |  |  |
| Yes (1) | 15 (93.8%) | 31 (73.8%) |  |  |
| **Consumption of red meat** |  |  | 1.000 |  |
| No (0) | 1 (6.25%) | 4 (9.50%) |  |  |
| Yes (1) | 15 (93.8%) | 38 (90.5%) |  |  |
| **Consumption of white meat** |  |  | 0.664 |  |
| No (0) | 2 (12.5%) | 4 (9.50%) |  |  |
| Yes (1) | 14 (87.5%) | 38 (90.5%) |  |  |
